# Complications and Ethical Challenges in Neurosurgery for Psychiatric Disorders

**DOI:** 10.3390/brainsci15121303

**Published:** 2025-12-02

**Authors:** Cristina V. Torres Díaz, Joaquín Luis Ayerbe Gracia, Mónica Lara Almunia, Gonzalo Olivares Granados, Marta Navas García, Paloma Pulido Rivas, Marta Del Alamo De Pedro, Rafael García De Sola, Álvaro Moleón-Ruiz

**Affiliations:** 1 Department of Neurosurgery, Hospital Universitario de La Princesa, Universidad Autónoma de Madrid, 28006 Madrid, Spain; martasoti@yahoo.es (M.N.G.); ppulido@neurorgs.com (P.P.R.); 2 Clinica Nuestra Señora Del Rosario, 28006 Madrid, Spain; rgsola@neurorgs.com; 3 Department of Neurosurgery, Fundación Jiménez Díaz University Hospital/Rey Juan Carlos, 41013 Madrid, Spain; jayerbe@fjd.es (J.L.A.G.); mncl23@gmail.com (M.L.A.); 4 Clinical Neurosurgery Unit, Hospital Universitario Virgen De La Victoria, 29010 Granada, Spain; goligra@msn.com; 5 Neurosurgery Department, University Hospital Ramón y Cajal, 28034 Madrid, Spain; marta_delalamo@yahoo.es; 6 Department of Psychiatry, Hospital Virgen del Rocío, Instituto Andaluz de Salud Cerebral, 41013 Sevilla, Spain; dr.alvaromoleon@gmail.com

**Keywords:** deep brain stimulation (DBS), psychiatric neurosurgery, treatment-resistant depression, obsessive–compulsive disorder, neuroethics, informed consent, cognitive side effects, neurosurgical complications, personal identity, multidisciplinary psychiatry

## Abstract

Functional neurosurgery has emerged as a potential therapeutic option for patients with severe, treatment-resistant psychiatric disorders such as obsessive–compulsive disorder (OCD) and major depressive disorder (MDD). Among the most studied interventions, deep brain stimulation (DBS) has shown promising outcomes in open-label studies, though controlled trials have yielded more modest results. This discrepancy, along with concerns about surgical risks, neuropsychiatric side effects, and ethical challenges, has limited the broader implementation of psychiatric neurosurgery. This review explores the clinical complications of DBS—including vascular events, infections, and hardware-related issues—as well as cognitive and behavioral changes such as hypomania, apathy, and impulse control disorders. Ethical concerns are also addressed, including the difficulty of obtaining truly informed consent and the potential impact on personal identity. The article emphasizes the need for multidisciplinary teams, dynamic consent models, standardized protocols, and high-quality clinical trials to ensure safe, ethical, and effective use of neurosurgical interventions in psychiatry. Ultimately, the responsible integration of functional neurosurgery into psychiatric care requires not only technical precision but also ethical rigor and patient-centered collaboration.

## 1. Introduction

Functional neurosurgery has emerged as a therapeutic alternative for patients with severe psychiatric disorders who do not respond to conventional treatments. Among the conditions that have been subject to neurosurgical interventions are obsessive–compulsive disorder (OCD) and treatment-resistant major depression (TRD), two highly disabling clinical entities that significantly affect patients’ quality of life and burden healthcare systems. Despite the development of multiple pharmacological, psychotherapeutic, and non-invasive neuromodulation strategies (such as transcranial magnetic stimulation and transcranial direct current stimulation), a considerable proportion of patients continue to experience refractory symptoms. This has driven the search for more invasive and targeted interventions [1,2,3,4,5].

In this context, techniques such as deep brain stimulation (DBS) have raised expectations, with open-label studies reporting response rates of up to 80% in patients with OCD or treatment-resistant depression [1,2,3,4,5]. However, results from randomized controlled trials have been more modest, leading to questions about the consistency and generalizability of the benefits observed in preliminary studies [6,7,8]. This discrepancy between initial findings and more rigorous data has limited the expansion of these therapies beyond experimental settings, despite growing interest in their clinical application. A major criticism of these randomized controlled trials is that their study design was fundamentally flawed. The blinded evaluation periods were conducted too early, at a time when the therapeutic effects of DBS had not yet fully emerged, and without adequately accounting for the well-documented microlesion or “insertion” effect that can transiently improve symptoms immediately after electrode implantation. As a result, the sham-controlled phases may have underestimated the true antidepressant potential of DBS, as demonstrated in other randomized-controlled trials [9,10,11,12].

The difficulty in replicating the robust long-term improvements seen in open-label studies within randomized controlled trials may be partly due to insufficiently accurate targeting in psychiatric DBS. These interventions often require extended periods of parameter adjustment to ensure that stimulation is delivered to the optimal anatomical pathways. Evidence increasingly suggests that outcomes in treatment-resistant depression and obsessive–compulsive disorder become more consistent and clinically meaningful when precision targeting strategies are employed. This shift in understanding has contributed to the FDA granting breakthrough designation for a new DBS trial that incorporates these advanced targeting methods [13,14,15].

Beyond efficacy, neurosurgery for psychiatric disorders presents a number of unique challenges that distinguish it from other areas of functional neurosurgery. Although psychiatric illnesses can, in theory, affect judgment, motivation, or perception of reality, there is no empirical evidence that typical candidates for DBS—such as patients with treatment-resistant depression or obsessive–compulsive disorder—systematically exhibit impaired decision-making capacity. On the contrary, existing studies consistently show that these patients retain intact autonomy and the ability to provide valid informed consent. Despite this, concerns about decisional vulnerability are often repeated in the literature without strong data to support them. At the same time, the absence of reliable biomarkers to predict treatment response continues to complicate candidate selection, increasing the risk of suboptimal outcomes or unnecessary adverse effects [16,17].

Ethical concerns in psychiatric neurosurgery often center on possible changes in personality, cognition, or identity after DBS. However, reports of persistent mood disturbances, cognitive decline, or dissociative symptoms lack solid empirical support. Most such effects are rare, transient, and reversible with stimulation adjustments, and there is no clear evidence that DBS alters personality beyond a return to premorbid functioning. Notably, randomized data in treatment-resistant depression show that postoperative cognitive changes resolve over time. Addressing these issues requires a multidisciplinary approach to ensure responsible clinical practice [8].

This article aims to analyze the specific outcomes and challenges associated with neurosurgery for the treatment of psychiatric disorders. It discusses surgical and neuropsychiatric risks, as well as the ethical, technical, and scientific barriers that currently limit widespread implementation. Through this critical review, we aim to contribute to the academic debate on the future role of neurosurgery in the management of severe mental illness.

## 2. General Complications of Neurosurgery in Psychiatric Disorders

Functional neurosurgery applied to psychiatric disorders—such as DBS for the treatment of OCD and treatment-resistant major depression—has shown encouraging results in subgroups of patients who have not responded to conventional therapies. Various studies have reported significant clinical improvements, both in affective symptoms and overall functioning, particularly in protocols carried out by highly experienced centers [1,2,3,4,5]. Nevertheless, these procedures involve a series of clinically relevant risks that must be carefully considered.

Complications associated with psychiatric neurosurgery can be grouped into two main categories: surgical and neuropsychiatric. Surgical complications reflect the risks inherent to any intracranial procedure, including hemorrhage, infection, electrode displacement, or hardware malfunction. Although the overall rate of major adverse events is low in experienced centers, commonly cited figures often derive from large cohorts of patients with Parkinson’s disease—typically older individuals with substantial medical comorbidity and higher baseline vulnerability [18,19,20,21,22,23,24,25,26,27,28,29,30]. As a result, these estimates may overstate the actual risk in psychiatric populations, who are generally younger and medically healthier [23,24,31].

On the other hand, neuropsychiatric complications are more complex and less predictable. Mood disturbances—such as hypotimia, hyperthymia, apathy, or abulia—as well as personality changes, cognitive complaints, or dissociative experiences have been described as potential side effects of chronic stimulation of circuits involved in emotion and motivation. However, current evidence indicates that these effects are generally transient and reversible with stimulation adjustments, and there is no robust data demonstrating that they persist over time or lead to lasting functional impairment [30,31].

A key challenge in this field is the limited availability of validated biomarkers that could help predict treatment response or anticipate adverse effects. While functional neurosurgery for conditions such as epilepsy or movement disorders benefits from long-standing selection and follow-up frameworks, psychiatric neurosurgery is still developing comparable tools to guide risk stratification and outcome optimization. Even so, progress has been significant in recent years. For example, there is now increasing consensus on how to define treatment refractoriness, with guidelines commonly describing treatment-resistant severe depression as failure to respond to at least two adequate antidepressant trials and that has been present for at least 5 years. These emerging standards help reduce uncertainty in surgical decision-making and contribute to greater consistency across clinical programs [32,33].

The very nature of psychiatric disorders has often been assumed to add an additional layer of complexity: symptoms could, in theory, affect a patient’s ability to understand and consent to the procedure, and clinical outcomes can be harder to quantify due to subjective variability and psychosocial influences. However, current evidence does not support the notion that patients with conditions such as treatment-resistant depression or obsessive–compulsive disorder are inherently less capable of informed decision-making than patients undergoing DBS for Parkinson’s disease, epilepsy, or chronic pain [16,17]. In this context, a cautious, multidisciplinary, and patient-centered approach remains essential—one that considers not only the immediate risks but also the long-term implications of intervening in neural circuits involved in mood, motivation, and behavior.

## 3. Surgical and Vascular Complications

**Intracerebral Hemorrhage (ICH):** The incidence of ICH associated with DBS ranges from 0.5% to 3% [19,21], depending on factors such as the center’s experience and the surgical technique used. Importantly, these figures come largely from cohorts dominated by patients with Parkinson’s disease, who are typically older and carry higher vascular and medical comorbidity than psychiatric populations. A retrospective study of 275 patients (527 procedures) reported an ICH incidence of 2.3%, identifying intraoperative systolic blood pressure as a significant risk factor (OR = 1.05; 95% CI: 1.01–1.09; *p* = 0.023) [19]. ICH in OCD patients implanted for DBS has been reported to arise in 2.2–2.6% of the cases [31].

**Infections:** Hardware-related infections are among the most common complications, with rates ranging between 2.8% and 9.95%. One study involving 426 patients reported an infection rate of 2.8% [20], while another study found an incidence of 9.95% in 201 patients [21].

**Hardware-related Complications:** These include skin erosion due to the presence of wires, migration or fracture of electrodes, and malfunction of the implantable pulse generator (IPG). In a study of 519 patients, hardware-related complications were observed in 6.7% of cases, with infection being the most frequent (2.95%), followed by electrode migration (0.6%) and cable fractures (0.26%). Some studies suggest that in patients with neuropsychiatric disorders, the complication rate tends to be slightly higher than in movement disorders, possibly due to increased compulsive manipulation of wounds and devices, the experimental nature of the procedures (which tend to be longer), or even molecular differences [20,22,24].

**Surgical Reinterventions:** Complications may require additional surgeries, such as IPG replacement due to recharging or interrogation issues, or complete system removal in cases of persistent infection. In the study of 426 patients, 6.3% required revision surgery due to complications [21].

It is important to highlight that complication rates tend to decrease with surgical team experience. In the study of 519 patients, the complication rate significantly dropped from 23% in the first 100 cases to 7% in the last 100 cases, demonstrating a positive learning curve [22] (Table 1).

## 4. Cognitive and Behavioral Complications of DBS in Psychiatric Disorders

In addition to the previously described surgical and technical risks, there are adverse cognitive and behavioral effects that require specialized clinical attention and close monitoring.

### 4.1. Cognitive Impairments

The cognitive effects of DBS in psychiatric disorders are variable and depend on the anatomical target, stimulation parameters, and individual patient characteristics. One of the most consistent findings in studies on DBS for OCD is a mild but significant decline in verbal fluency and processing speed, particularly when stimulating the nucleus accumbens [34]. Working memory impairments have also been observed, especially in tasks requiring sustained attention and active manipulation of information. These effects, however, tend to be transient or manageable through adjustments in stimulation parameters [35].

In contrast to these observations, more recent research has challenged the notion that DBS is exclusively associated with cognitive decline. A recent meta-analysis on DBS for treatment-resistant depression found small but significant improvements in verbal memory, visual memory, attention/psychomotor speed, and executive functioning after 6 to 18 months of stimulation [35].

Moreover, a study by Grubert et al. assessed the neuropsychological safety of nucleus accumbens DBS in patients with treatment-resistant major depression and found significant improvements in attention, learning and memory, executive functions, and visual perception after 12 months of continuous stimulation [24].

### 4.2. Behavioral and Emotional Changes

#### 4.2.1. Episodes of Hypomania and Euphoria

Manic-like episodes, especially hypomania, have been reported. These are characterized by increased speech, impulsivity, hyperactivity, and impaired social judgment, particularly in patients receiving stimulation of the ventral internal capsule or the nucleus accumbens. These effects are usually transient and, in some cases, reversible through adjustments in stimulation parameters [36].

#### 4.2.2. Impulse Control Disorders

Impulse control disorders—such as compulsive behaviors, excessive shopping, hypersexuality, and gambling—have been documented, particularly when the stimulation target involves subcortical structures of the reward system, such as the subthalamic nucleus or the nucleus accumbens. These adverse effects can often be managed through stimulation adjustments and psychotherapeutic support [16]. Such neuropsychiatric changes are frequently reported in the context of subthalamic DBS for Parkinson’s disease—a target that has also shown efficacy in treating OCD—although the stimulation site used for OCD is located more medially within the subthalamic region than the site typically targeted for movement-related Parkinson’s disease [8,17,18,19]. Parkinson’s disease is fundamentally different from OCD or depression, and the psychiatric manifestations observed during DBS for Parkinson’s disease can be influenced by multiple factors, including aging, disease progression and neurodegeneration, the specific stimulation target, programming parameters, and postoperative medication adjustments [25,26,27,28,29].

#### 4.2.3. Changes in Personality and Identity

Although modulation of deep brain networks through DBS can, in some cases, be associated with changes in personality traits or a subtle shift in a patient’s sense of identity, such effects are not the norm and are typically mild, transient, and manageable. Many patients describe these experiences not as negative transformations but as adjustments linked to symptom improvement, medication reduction, or adaptation to restored function. When present, these psychological changes can be effectively addressed through careful monitoring, adjustment of stimulation parameters, and supportive follow-up. Importantly, one study highlighted the ethical value of proactively considering potential effects of DBS on personal identity, underscoring the need for thoughtful, patient-centered assessment rather than indicating frequent or severe alteration [20] (Table 2).

## 5. Ethical and Scientific Considerations in Neurosurgery for Psychiatric Disorders

Finally, the development and clinical implementation of functional neurosurgery for psychiatric disorders must be grounded in rigorous empirical evidence that combines robust clinical outcomes with international ethical and methodological standards.

Unlike other areas of functional neurosurgery—such as Parkinson’s disease or epilepsy—where the efficacy of interventions like DBS has been extensively validated through multicenter clinical trials and meta-analyses, the field of psychiatry is still in a phase of scientific consolidation [37,38,39,40,41,42].

Initial open-label studies of DBS for disorders such as treatment-resistant depression (TRD) or OCD showed encouraging clinical response rates, with significant reductions on symptom scales such as the Hamilton Depression Rating Scale (HDRS) and the Yale-Brown Obsessive Compulsive Scale (Y-BOCS). In some cases, more than 50% of patients met criteria for remission or sustained clinical improvement [1,2,3,4,5].

However, controlled clinical trials designed to rigorously assess the efficacy of DBS under double-blind, placebo-controlled conditions have faced numerous methodological challenges. These include the microlesion effect, which can induce transient improvement even before stimulation is activated; delayed clinical response, which may take weeks or months to emerge; and the difficulty of establishing homogeneous response criteria in highly heterogeneous populations. These difficulties have led some pivotal trials, such as the one by Mayberg et al. on subgenual cingulate stimulation for TRD, to be discontinued before reaching statistical significance during the blinded phase—despite observing substantial improvements during open-label follow-up [43].

Similarly, the multicenter trial led by Holtzheimer et al., which also investigated subgenual cingulate DBS, failed to demonstrate statistically significant superiority over placebo during the 16-week double-blind phase. However, as in Mayberg’s study [44], a considerable proportion of patients experienced clinically relevant improvements once the blind was lifted and active stimulation began [42].

In the case of OCD, the controlled evidence has been more robust, with randomized double-blind studies providing Class I evidence of efficacy [43]. In the trial by Mallet et al., stimulation of the subthalamic nucleus yielded statistically significant differences between the active and sham phases, with a substantial reduction in obsessive–compulsive symptoms [34]. Other open-label studies have reported sustained improvements over time, with response rates exceeding 50% after 12 months of stimulation [40].

Currently, several studies are underway to overcome previous limitations and provide more definitive data on the efficacy and safety of DBS in psychiatry. One notable example is the TRANSCEND trial (Treatment ResistAnt Depression Subcallosal Cingulate Network Deep Brain Stimulation), a randomized, controlled, and longitudinal study that uses a crossover design and individualized connectomic analysis to optimize target and candidate selection [15].

Another promising trial is FORESEE III, which investigates stimulation of the superolateral medial forebrain bundle (slMFB) in patients with treatment-resistant depression. This study integrates advanced imaging techniques and individualized connectivity-based planning to personalize the intervention and maximize therapeutic impact [44].

Although compassionate use of DBS in psychiatry may be considered in some circumstances—particularly when all conventional therapeutic alternatives have been exhausted and the patient is experiencing severe, refractory suffering—the preferred and most ethical path remains participation in structured clinical trials. Even in N = 1 studies with rigorous methodological design, systematic follow-up, ethical oversight, clinical analysis, and meaningful contribution to collective knowledge are ensured.

In this regard, the development of psychiatric neurosurgery should not be understood merely as a technical advancement, but as a joint ethical and scientific endeavor that demands transparency, rigor, caution, and a strong commitment to patient protection and the generation of generalizable knowledge.

## 6. Discussion

Functional neurosurgery, and particularly DBS, entails risks inherent to any invasive neurosurgical intervention, including vascular complications (such as intracranial hemorrhage), infections, and hardware-related issues. Although these complications are relatively rare, they can have significant clinical impact. These general risks, widely documented in the literature, should serve as the starting point for assessing the overall safety of such interventions in psychiatric disorders [31].

Beyond these surgical and technical risks, neurosurgery for psychiatric disorders presents additional challenges in the cognitive and behavioral domains. It is well known that modulation of deep brain circuits involved in emotion, motivation, and executive functions can induce changes in cognitive performance as well as in behavior and emotional state. In this sense, DBS in patients with disorders such as OCD or treatment-resistant depression may lead to mild decreases in verbal fluency and processing speed, although some studies have also reported improvements in episodic memory and attention [45,46]. Behaviorally, diverse phenomena such as manic episodes, depressive symptoms, and impulse control disorders have been documented, reflecting the complexity of the intervention and the need for fine-tuning the stimulation to maximize benefits and minimize adverse effects [36].

These neuropsychological complications, while particularly relevant in psychiatry, are not exclusive to this field. For example, in Parkinson’s disease, subthalamic nucleus and globus pallidus DBS is also associated with risks of deterioration in verbal fluency and executive functions, as well as emotional changes including apathy and impulse control disorders [47,48,49,50,51]. In epilepsy surgery—especially after temporal lobectomies—verbal memory decline and other cognitive deficits are common, which may worsen in patients with extensive resections or advanced age [52,53,54,55]. In chronic pain settings, surgical interventions may provoke alterations in body perception, autonomic dysfunction, and emotional changes, underscoring that any neurosurgical intervention affecting complex circuits may trigger multifaceted effects [56].

From an ethical standpoint, obtaining informed consent represents a particularly significant challenge in psychiatric neurosurgery due to the cognitive and emotional difficulties inherent to this patient population. These require rigorous assessments of decisional capacity and a meticulous process of information delivery and support [16,17,56,57]. Likewise, concerns about potential alterations in personal identity—although understandable—are tempered by clinical evidence suggesting a recovery of the “authentic self” following symptom reduction, rather than a profound personality change [58]. It is therefore necessary to implement a dynamic and ongoing model of informed consent, rather than a single formal moment before surgery. This model should include periodic evaluations of decisional capacity, delivery of information adapted to the patient’s cognitive level, and the active involvement of family members and mental health professionals. Such an approach provides emotional and contextual support throughout the process, reinforcing autonomy without sacrificing ethical protection.

As for postoperative follow-up, the need for personalized and interdisciplinary monitoring is crucial. This follow-up should not be limited to neurological or surgical visits, but must also include ongoing psychiatric and neuropsychological assessments, with the ability to respond promptly to neuropsychiatric side effects such as hypomania, apathy, or impulse control disorders. Ideally, these teams should be coordinated and protocolized, with clear referral structures and stimulation adjustment procedures when relevant behavioral changes are detected [1,2,3,4,5,6,7,8,9,10]. Regarding concerns about potential alterations in personality or identity, it is essential to incorporate an ethical perspective from the earliest stages, through the involvement of specialized committees conducting ongoing evaluations. Additionally, the patient’s voice must be actively present throughout the entire therapeutic process, both in decision-making and in outcome assessment. Rather than being seen as an inevitable negative side effect, this dimension should be approached as an opportunity to restore agency and authenticity in individuals whose lives have been dominated by severe symptoms [10,20,57,58,59].

One factor that continues to limit progress in the field of functional neurosurgery for psychiatric disorders is the absence of reliable biomarkers that could predict clinical response or the risk of complications. This limitation, which is shared with other areas such as chronic pain management, complicates the precise selection of candidates and the personalization of treatment—thereby increasing uncertainty and the need for close monitoring and well-designed clinical studies [59]. Moreover, there is generally no unequivocal consensus on how to define treatment refractoriness in these disorders. The absence of predictive biomarkers necessitates reliance on detailed clinical assessments, but accuracy could be enhanced through the adoption of standardized protocols combining clinical, neuropsychological, and advanced neuroimaging tools. Validating these protocols in multicenter settings would not only improve patient selection but also help establish objective criteria for defining “refractoriness” to conventional treatments—currently a variable and poorly standardized concept.

At the scientific level, a key limitation in the field remains the lack of controlled studies with sufficient statistical power and methodological rigor. To make progress, it is essential to promote collaborative, multicenter, well-designed clinical trials that overcome previous shortcomings and provide more conclusive evidence on the efficacy and safety of DBS in psychiatry. The creation of national registries—ideally integrated into platforms such as REDCap, like https://www.clinicaltrials.gov/study/NCT02071134 (accessed on 1 November 2025)—would facilitate the systematic collection of data, even in centers with small case numbers, contributing to collective knowledge and the continuous improvement of clinical practice.

Finally, it is important to reinforce the idea—already introduced earlier—that addressing the ethical and technical challenges in psychiatric neurosurgery requires a multidisciplinary, patient-centered approach supported by the specific training of involved teams. This includes not only technical and clinical expertise but also communication skills, ethical sensitivity, and the ability to work collaboratively— without which quality care for this vulnerable population would not be possible.

**Figure 1:**
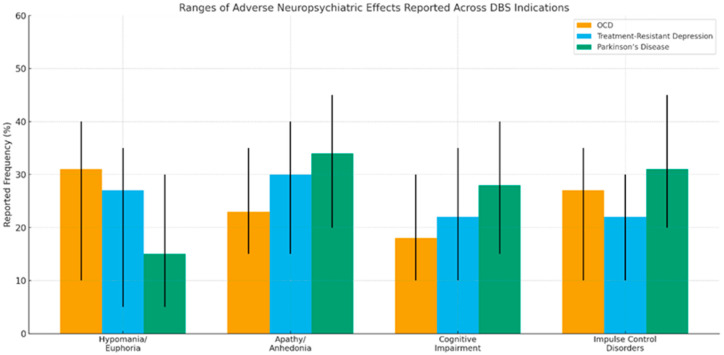
Comparison of the relative frequency of adverse neuropsychiatric effects reported in deep brain stimulation (DBS) for obsessive–compulsive disorder (OCD), treatment-resistant depression (TRD), and Parkinson’s disease (PD). Bars represent the representative frequencies used in this narrative review. Vertical error bars indicate the approximate minimum–maximum range reported across clinical studies for each indication, rather than standard deviations or confidence intervals, as no pooled quantitative meta-analysis was performed. Ranges were extracted from prospective cohorts, randomized controlled trials, and systematic reviews reporting neuropsychiatric outcomes in DBS for OCD, TRD and PD.

In summary, functional neurosurgery offers significant therapeutic opportunities but must be implemented with caution—always considering the general surgical risks, the specific cognitive and behavioral complexities of each disorder, and the current limitations in outcome prediction. The implementation of specific, personalized protocols and a multidisciplinary approach are key to advancing toward a safe, ethical, and effective practice that optimizes quality of life for patients with refractory neuropsychiatric disorders (Figure 1).

## 7. Conclusions

Functional neurosurgery—particularly deep brain stimulation—represents a promising therapeutic alternative for patients with severe and treatment-resistant psychiatric disorders. However, its implementation poses clinical, ethical, and methodological challenges that must be addressed in a rigorous and structured manner.

Although surgical and neuropsychiatric complications are relatively infrequent in experienced hands, they require careful risk–benefit assessment and sustained interdisciplinary follow-up. Furthermore, the variability in cognitive and behavioral effects highlights the need for individualized adjustment of stimulation parameters and specialized clinical supervision.

From an ethical standpoint, it is essential to adopt a dynamic informed consent model, tailored to the cognitive and emotional profile of each patient, and involving both family members and mental health professionals in the decision-making process.

Likewise, the early inclusion of ethics committees and the active voice of the patient are essential to preserve autonomy and a sense of identity.

In parallel, the lack of reliable biomarkers and the absence of uniform protocols for defining treatment refractoriness hinder the appropriate selection of candidates. In this regard, the validation of combined diagnostic tools, as well as the promotion of collaborative, multicenter, and methodologically robust clinical trials, is necessary.

Finally, all progress in this discipline must be supported by the specific training of the teams involved, encompassing not only technical competence but also ethical sensitivity and communication skills. Only through a truly multidisciplinary, patient-centered, and evidence-based approach will it be possible to consolidate a safe, effective, and ethically responsible neurosurgical practice in the field of psychiatric disorders.

## Figures and Tables

**Table 1:** Surgical complications associated with deep brain stimulation (DBS).

Type of Complication	Incidence (%)	Main Observations
Intracerebral hemorrhage (ICH)	2.2–2.6%	Increased risk with elevated intraoperative systolic blood pressure [31]
Hardware-related infections	0–9.95%	Higher risk in psychiatry due to compulsive manipulation and protocol duration [20,21]
Hardware complications	Up to 6.7%	Electrode migration, cable fracture, IPG malfunction [20,22,24]
Surgical reinterventions	Up to 6.3%	Due to infections, IPG failure, or technical complications [21,22]

Source: Compilation of data from multicenter clinical studies on DBS complications [20,21,22,23,24,31].

**Table 2:** Adverse neuropsychiatric effects reported after DBS.

Type of Effect	Main Characteristics	Frequency/Observations
Cognitive impairments	Decreased verbal fluency and processing speed	Potentially reversible with parameter adjustments. Common with nucleus accumbens stimulation [35]
Hypomania/euphoria	Impulsivity, hyperactivity, impaired social judgment	Reversible with parameter changes. Ventral internal capsule or nucleus accumbens stimulation [36]
Apathy/anhedonia	Reduced emotional reactivity	Reversible with parameter changes. Excessive stimulation of limbic structures [20,22,23,24,25]
Impulse control disorders	Compulsive shopping, hypersexuality, gambling	Reversible with parameter changes. Stimulation of reward system structures [20,22,23,24,25]
Personality/identity changes	Feeling “different,” altered sense of self	Requires rigorous ethical evaluation [20]

Source: Adapted from clinical reviews and meta-analyses in patients with DBS for OCD and treatment-resistant depression [20,22,23,24,25,35,36].

## Data Availability

No new data were created or analyzed in this study.
